# Silibinin Inhibits NSCLC Metastasis by Targeting the EGFR/LOX Pathway

**DOI:** 10.3389/fphar.2018.00021

**Published:** 2018-02-08

**Authors:** Xiaoying Hou, Hongzhi Du, Xingping Quan, Lei Shi, Qianqian Zhang, Yao Wu, Yang Liu, Jing Xiao, Yong Li, Ligong Lu, Xun Ai, Meixiao Zhan, Shengtao Yuan, Li Sun

**Affiliations:** ^1^Jiangsu Key Laboratory of Drug Screening, China Pharmaceutical University, Nanjing, China; ^2^Jiangsu Center for Pharmacodynamics Research and Evaluation, China Pharmaceutical University, Nanjing, China; ^3^Henan Key Laboratory of Organic Functional Molecule and Drug Innovation, School of Chemistry and Chemical Engineering, Henan Normal University, Xinxiang, China; ^4^School of Pharmaceutical, Lanzhou University, Lanzhou, China; ^5^Center of Intervention Radiology, Zhuhai Precision Medicine Center, Zhuhai People’s Hospital, Zhuhai, China; ^6^Department of Molecular Biophysics and Physiology, Rush University Medical Center, Chicago, IL, United States

**Keywords:** lysyl oxidase, EGFR, silibinin, NSCLC, metastasis

## Abstract

Tumor metastasis is the most lethal and debilitating process that threatens cancer patients. Among the regulators involved in tumor metastasis, lysyl oxidase (LOX) is an important contributor for tumor invasion, migration and the formation of the pre-metastatic niche. Although the relationship between LOX and poor prognosis of lung patients has been preliminary reported, the mechanism remains poorly understood. Here, we found that LOX overexpression is closely related to the survival of lung adenocarcinoma patients but not squamous cell carcinoma patients. Moreover, we confirmed that LOX expression is regulated by the activation of epidermal growth factor receptor (EGFR) via the PI3K/AKT, MEK/ERK, and SAPK/JNK signaling pathways in non-small cell lung cancer (NSCLC). Meanwhile, the study also suggested that the traditional anti-fibrosis drug silibinin inhibited NSCLC cell migration in an EGFR/LOX dependent manner. In addition, an orthotopic implantation metastasis model also confirmed that the EGFR inhibitor WZ4002 and silibinin decreased tumor metastasis through the EGFR/LOX pathway. Altogether, this study revealed that LOX expression is regulated by the EGFR pathway and this may account for the anti-cancer metastasis effects of silibinin, indicating LOX as a potentially therapeutic target for NSCLC treatment.

## Introduction

Tumor metastasis is the most lethal and debilitating process that threatens cancer patients. Numerous studies fully confirmed that metastasis is a systemic process involving a variety of cells and microenvironments (Bohn et al., 2017). Therefore, an increasing number of researchers are paying attention to tumor metastasis as well as tumor proliferation. Last decade, we have been engaged in tumor metastasis research, to find potential anti-cancer metastasis targets and drugs (Lin et al., 2009, 2012; Zhou et al., 2011; Wei et al., 2016; Du et al., 2017a). Recently, based on bioinformatics study, we found lysyl oxidase (LOX) overexpression is closely related to the survival of lung adenocarcinoma patients but not squamous cell carcinoma patients. As previously reported, LOX may be an important regulator in cancer cells migration, invasion and the formation of a mature extracellular matrix which promotes tumor progression (Wang et al., 2017). The LOX family consists of LOX and four LOX homologues (LOXL1, LOXL2, LOXL3, and LOXL4), all of them share a highly conserved carboxyl terminal domain and diverse amino terminal regions (Cox et al., 2013; Trackman, 2016). Among the LOX family, the function of LOXL1, LOXL3 and LOXL4 is poorly understood, while evidence shows that overexpression of LOX and LOXL2 may be involved in the multiple steps of tumor metastasis and contributes to cancer progression (Peng et al., 2017). LOX is related to the poor prognosis in breast cancer (Chu et al., 2012), lung cancer (Liu et al., 2014), colorectal cancer (Nebuloni et al., 2016), uveal melanoma (Abourbih et al., 2010), pancreatic cancer (Miller et al., 2015), gastric cancer (Zhang et al., 2013), and laryngeal cancer (Lee et al., 2017). However, it has only been reported that LOX expression is regulated by HIF-1α under hypoxia (Rankin and Giaccia, 2016) and is induced by TGF-β (Fang et al., 2016) in cancer cells. But beyond that, the regulatory mechanism of LOX needs more exploration.

Lung cancer is the most common incident cancer and the leading cause of cancer death in China (Chen et al., 2016). Meanwhile, half of the lung adenocarcinoma patients in Asia are accompanied with EGFR mutation (Midha et al., 2015). EGFR targeted therapy brings hope for lung adenocarcinoma patients especially non-small cell lung cancer (NSCLC) patients. Unfortunately, the EGFR inhibitors widely face primary resistance (approximately 60%) and rapidly generate acquired resistance (6 to 12 months) (Eberlein et al., 2015). Therefore, it is urgent to find a downstream effector of the EGFR, as a substitutable therapeutic target of the EGFR, to overcome the drug resistance. The previous study showed that EGFR inhibition attenuates liver fibrosis (Komposch and Sibilia, 2016) and the development of hepatocellular carcinoma (Berasain and Avila, 2014). Given that LOX is a key enzyme accounting for fibrosis, it is interesting to explore whether EGFR regulates LOX and whether LOX acts as a therapeutic target for NSCLC.

Currently, chemotherapy, surgery, radiotherapy, biotherapy and other adjuvant therapies are the common strategies for treating cancer. Chemotherapy has always been the preferred and the most significant treatment for NSCLC patients (Redondo-Blanco et al., 2017). However, almost all drugs easily acquire resistance after a period of treatment, such as the T790M mutation conferring resistance to first-generation EGFR TKIs (Tong et al., 2017). Therefore, it is urgent to find new effective antitumor drugs. An increasing number of researchers all over the world are focused on natural medicines or drug repurposing to find new antitumor drugs (Redondo-Blanco et al., 2017). Silibinin is a natural polyphenolic flavonoid isolated from seed extracts of the herb milk thistle (Silybum marianum). It is a classical drug for hepatic fibrosis in clinical in many countries and regions. Interestingly, abundant studies and clinical trials^1^ suggesting that silibinin exhibits anti-cancer effect, but the mechanism is incompletely understood (Wu et al., 2013; Bosch-Barrera et al., 2016). As an anti-fibrotic drug, whether silibinin inhibiting metastasis via LOX requires further exploration.

Altogether, this study confirmed that LOX expression is regulated by EGFR *in vitro* and *in vivo*. It also showed that the EGFR/LOX pathway may play a role in the anti-metastasis effects of silibinin in NSCLC, which provides a scientific basis for developing silibinin into an anti-cancer drug. Importantly, LOX may be a potential target for medicinal development in the clinic.

## Materials and Methods

### Database

The free survival of lung cancer patients with different LOX gene expression was analyzed using Kaplan-Meier Plotter^2^. The expression of EGFR and LOX in human tumor tissue was analyzed by the Oncomine^TM^ database^3^. The correlation of EGFR and LOX gene expression in human NSCLC tissue was performed by TCGA Research Network^4^.

### Cell Culture

Human lung cancer cell lines, NCI-H1975, HCC827, A549, H460, and NCI-H1299 were obtained from the Cell Bank of the Institute of Biochemistry and Cell Biology, Chinese Academy of Sciences (Shanghai, China). The cells were cultured in RPMI-1640 (Gibco) with 10% Foetal Bovine Serum (HyClone). All the lung cancer cells were recently authenticated by short tandem repeat (Genetic Testing Biotechnology Corporation, Suzhou) and were tested for mycoplasma contamination.

### Human Tumor Tissue Array

Human lung cancer specimens were collected from the National Engineering Center for Biochip at Shanghai (SBC), China. The study was approved by the Research Ethics Committee in Taizhou Hospital of Zhejiang Province and written informed consent was obtained from all participants. In total, 30 patients who underwent surgery for histologically proven lung adenocarcinoma were selected in this research. There were a total of 14 men and 16 women, whose ages range from 37 to 71 years.

### Antibodies and Reagents

The antibodies and reagents included EGFR (4267s, CST), P-EGFR (Y1068) (3777S, CST), LOX (A10960, ABclonal), AKT (9272S, CST), P-AKT (S473) (#4060, CST), ERK1/2 (4695S, CST), P-ERK1/2 (Thr202/Tyr204) (4370s, CST), P38 MAPK (8690S, CST), P-P38 MAPK (T180-Y182) (4511S, CST), SAPK/JNK(56G8) (9258S, CST), P-SAPK/JNK (T183/Y185) (4668S, CST), LY294002 (S1105, Selleck), U0126 (S1102, Selleck), SB203580 (S1076, Selleck), SP600125 (S1460, Selleck), and TGF-α(Pepro, Tech).

### Western Blot

The total cell lysates were extracted with lysis buffer containing protease and phosphatase inhibitors. The proteins were fractionated by 6–15% sodium dodecyl sulfate polyacrylamide gel electrophoresis (SDS-PAGE) and were transferred to PVDF membrane (Millipore, United States). The membranes were blocked with 5% BSA-TBST for 1 h at room temperature and were incubated with primary antibodies (diluted in 5% BSA-TBST) for 18 h at 4°C. Next, they were probed with secondary antibodies for 1 h at room temperature. The expression of the target proteins was detected by the Immobilon Western chemiluminescent HRP Substrate (Millipore, United States). Quantification of protein bands was carried out using densitometry and the respective densitometry graphs are shown in the Supplementary Figures.

### RT-PCR

Total cellular RNA was isolated with the TRIzol^®^ Reagent (Vazyme) and was reverse transcribed with the HiScript^TM^ QRT SuperMix for qPCR (Vazyme). The mRNA level was measured with the SYBR Green master mix (Vazyme). The amount of mRNA for each gene was standardized with the internal control (18s mRNA). Each treatment group was compared with the control group to show the relative mRNA level.

### siRNA Transfection

All the siRNA were synthesized by GenePharma. The cells were transiently transfected using Lipofectamine^TM^ 2000 (Invitrogen) according to the manufacturer’s recommendations.

### Cell Proliferation Assays

The cell proliferation assays were performed by MTT. The cells were seeded into 96-well plates in 180 μL of medium per well. After 12 h, the cells were treated with silibinin at specified concentrations for additional 12, 24, 48, or 72 h. The cells were treated with 5 mg/mL of MTT solution (20 μL/well) for 4 h. Then, the supernatant was replaced and DMSO (150 μL/well) was added. After the formazan was dissolved, the absorbance was measured at 570 nm using a Universal Microplate Reader (EL800; Bio-tek Instruments Inc.). The inhibitory ratio was calculated by the following formula: inhibitory ratio (%) = (1–average absorbance of treated group/average absorbance of control group) × 100. The results are presented as mean standard deviation (SD). In addition, triplicate experiments were performed in parallel.

### Migration Assay

The migration assay was detected by 24 Well Transwell Chambers (Millipore, United States). The cells were seeded at a density of 6–8 × 10^4^ cells per chamber with drugs at specified concentrations both in the lower and the upper chamber. The cells were allowed to migrate for 16 h. After discarding the medium, the migrating cells were stained with DiffQuik Stain Set (Jiancheng Bioengineering Institute), photographed, and counted with Image-Pro Plus software.

### Wound Healing Assay

Wound healing assay was carried out to determine the migration ability of the tumor cells. The cells were seeded into 6-well plates and incubated in complete medium to 90% confluence. A sterilized pipette tip was used to generate wounding across the cell monolayer, and then, the cells were washed twice with PBS, replaced with fresh media and treated with drugs at specified concentrations for another 12 h. The cells migrated into the wounded area were visualized and photographed under the inverted microscope.

### Molecular Docking

Schrödinger was used for the molecular modeling studies. The crystal structure of the EGFR kinase domain (PDB ID: 3W2S) was prepared using Protein Preparation Wizard. The structure of silibinin (ZINC02033589) was prepared using LigPrep. The calculation was performed based on the force field OPLS (optimized potentials for liquid simulations) 2005 choosing water as the solvent. The following structure was obtained from the result of 1000 calculation cycles.

### Metastasis Model of Lung Orthotopic Implantation

Five to six-week-old female BALB/c mice (18 to 22 g) were supplied by the Model Animal Research Center of Nanjing University. NCI-H1975 cells were collected in serum-free medium and were mixed with same volume Matrigel (Coring). The mice were anesthetized by isoflurane inhalation, and then, 50 μL of cell suspensions (1 × 10^6^) were orthotopically injected through the intercostal space into the lung immediately after making a small skin incision. The mice were randomly divided into four groups (6 mice/group) 11 days after the injection. Silibinin (70 mg/kg, 140 mg/kg) and WZ4002 (25 mg/kg) were administered intragastrically in the treatment groups daily for 3 weeks, while the control animals were administered the same vehicle. After 3 weeks, the metastatic nodes were detected by gross examination. The study was conducted in accordance with the standards established by the Experimental Animal Care Commission in China Pharmaceutical University.

### Immunohistochemistry

The P-EGFR and LOX levels in the lung tumors were evaluated by IHC using P-EGFR and LOX antibodies. The tumor tissues from the nude mice were formalin fixed and paraffin embedded. The paraffin embedded tissues were de-paraffinized, rehydrated, rinsed and immersed using 10 mM sodium citrate (pH 6.0). After treatment with methanol containing 3% hydrogen peroxide, the tissues were staining with P-EGFR, LOX, and secondary antibodies.

### Statistical Analysis

The relationship between EGFR and LOX expression was analyzed via the Spearman rank correlation test. The results were presented as the mean ± SD from triplicate experiments performed in a parallel manner unless otherwise indicated. Statistically significant differences were determined using GraphPad Prism 6 software. A value of *P* < 0.05 was considered significant.

## Results

### High Expression of LOX Is Associated with the Poor Prognosis of Lung Cancer Patients and the Phosphorylation of EGFR

To test the relationship between LOX expression and the prognosis of cancer patients, we used Kaplan–Meier plotter^5^ to analyze the correlation.

**FIGURE 1:**
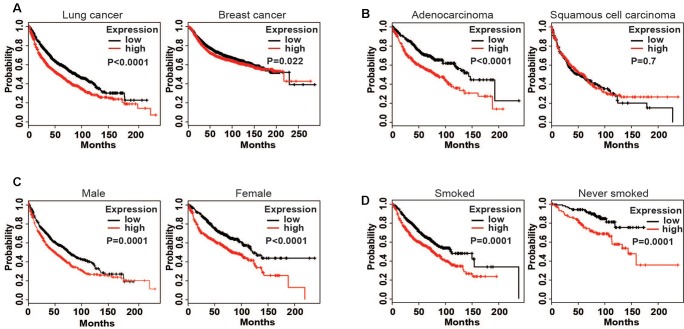
High expression of lysyl oxidase (LOX) is closely associated with the poor prognosis of lung cancer patients. Kaplan–Meier (http://kmplot.com/ analysis) was used to analyze the relapse-free survival of cancer patients. **(A)** LOX expression in lung cancer patients (*n* = 1926) and breast cancer patients (*n* = 3951). **(B)** LOX expression in lung adenocarcinoma patients (*n* = 720) and lung squamous carcinoma patients (*n* = 524). **(C)** LOX expression in male lung adenocarcinoma patients (*n* = 1100) and in female lung adenocarcinoma patients (*n* = 715). **(D)** LOX expression in smoked lung adenocarcinoma patients (*n* = 820) and in never smoked lung adenocarcinoma patients (*n* = 205).

**FIGURE 2:**
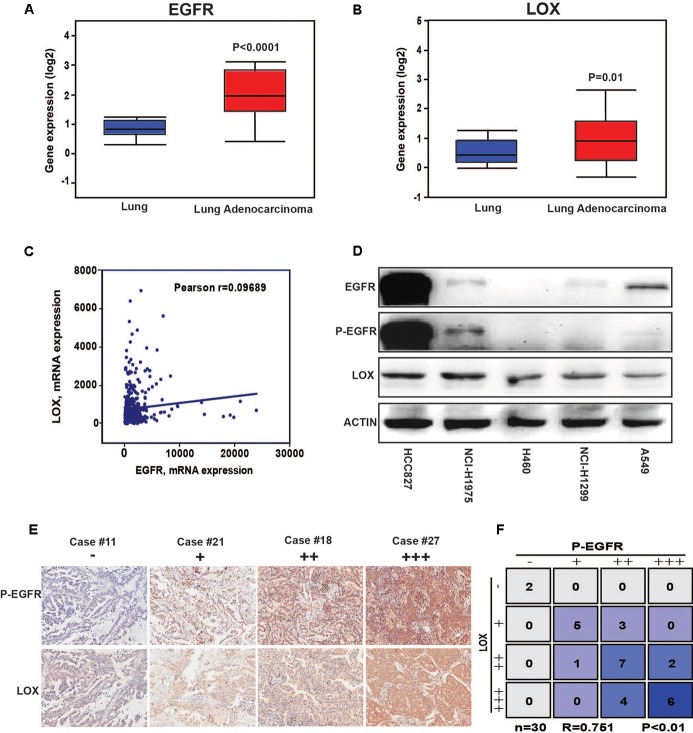
The expression of LOX is associated with the phosphorylation of epidermal growth factor receptor (EGFR) in non-small cell lung cancer (NSCLC). **(A)** EGFR expression levels in lung adenocarcinoma tissues and in normal tissues according to the Oncomine^TM^ database. **(B)** LOX expression levels in lung adenocarcinoma tissues and in normal tissues according to the Oncomine^TM^ database. **(C)** The correlation of EGFR and LOX gene expression in human NSCLC tissue was performed by TCGA Research Network (http://cancergenome.nih.gov). **(D)** Western blot of the EGFR, P-EGFR and LOX expression levels in the NSCLC cell lines (HCCC827, NCI-H1975, H460, NCI-H1299, and A549). **(E,F)** P-EGFR and LOX expression in tissue array of NSCLC patients (*n* = 30).

The results showed that a high expression of LOX was closely related to the poor prognosis of lung cancer patients (**Figure 1A**), and was more associated with lung adenocarcinoma patients but not lung squamous cell carcinoma patients (**Figure 1B**). In further exploration, data showed that influence of LOX expression on the survival of lung adenocarcinoma patients is independent of gender and smoking history (**Figures 1C,D**). As is reported, half of the lung adenocarcinoma patients were accompanied with an EGFR mutation (Midha et al., 2015). Thus, we suspected a relationship between EGFR and LOX expression. Firstly, according to the Oncomine^TM^ database^6^, the expression levels of EGFR and LOX were higher in lung adenocarcinoma tissues (**Figures 2A,B**). Furthermore, TCGA Research Network^7^ also indicated that there might be a correlation between EGFR and LOX mRNA expression in lung adenocarcinoma patients (**Figure 2C**). In fact, both the overexpression and mutation of EGFR eventually led to the phosphorylation and concomitant activation of downstream signaling, promoting the proliferation, metastasis, and resistance to chemotherapy in cancer cells. Therefore, we tested the expression of P-EGFR and LOX in five NSCLC cell lines, the data showed that expression of LOX was relatively higher in the cell lines with high expression of p-EGFR (NCI-H1975 and HCC827) than that with low P-EGFR expression (H460, NCI-H1299, and A549) (**Figure 2D** and **Supplementary Figure S1**). Moreover, human tumor tissue microarray further confirmed the possible correlation between P-EGFR and LOX (**Figures 2E,F**). Conclusively, these data suggest that there maybe a regulation relationship between P-EGFR and LOX expression.

### LOX Expression Is Regulated by the Phosphorylation of EGFR

**FIGURE 3:**
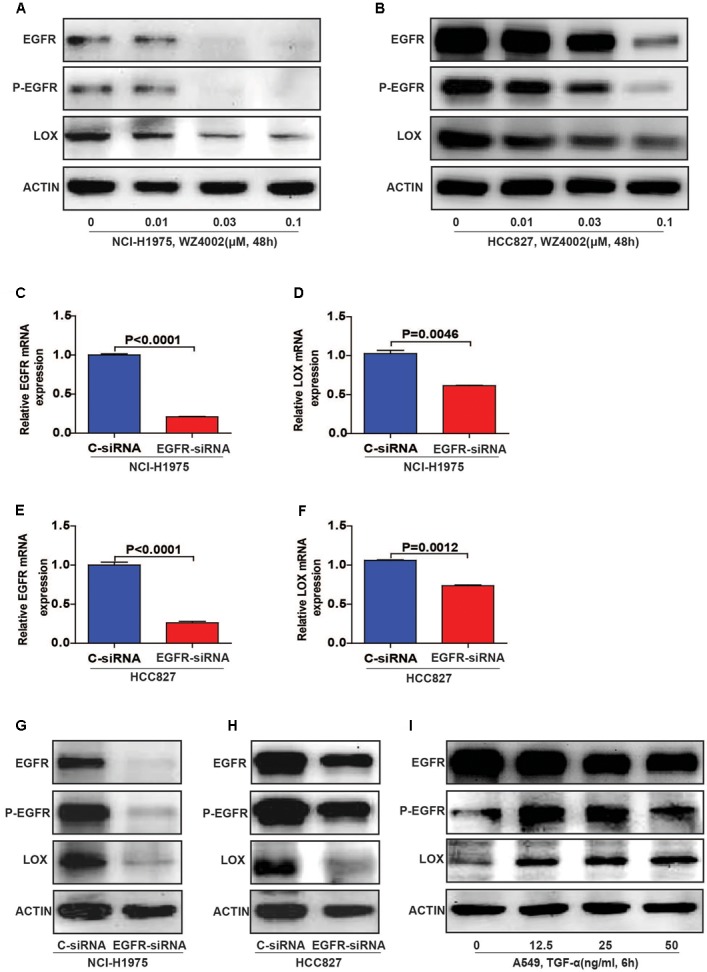
Lysyl oxidase expression is regulated by the phosphorylation of EGFR in NSCLC cell lines. **(A,B)** LOX levels decreased after the NCI-H1975 and HCC827 cells were exposed to the EGFR inhibitor WZ4002 for 48 h. **(C–H)** The knockdown of EGFR inhibited the expression of LOX at both the mRNA and protein levels in NCI-H1975 and HCC827 cell lines. **(I)** TGF-α (EGFR agonist) treatment for 6 h induced LOX expression in A549. The data are represented as the mean ± SD of three independent experiments. The *P*-values <0.05 were considered statistically significant for all the tests.

To further explore the relationship, we inhibited EGFR by WZ4002 in NCI-H1975 and HCC827 cells. Interestingly, the expression of LOX was also dramatically inhibited (**Figures 3A,B** and **Supplementary Figures S2A,B**). Meanwhile, when EGFR was knocked down in two NSCLC cells, the expression of LOX was remarkably decreased at both the mRNA and protein levels (**Figures 3C–H** and **Supplementary Figures S2C,D**). In addition, the phosphorylation of EGFR and LOX expression in EGFR wild type NSCLC cell line A549 were both up-regulated when treated with the EGFR agonist TGF-α. (**Figure 3I** and **Supplementary Figure S2E**). Here, we concluded that LOX expression is regulated by the phosphorylation of EGFR in NSCLC cell lines, but the mechanism needs to be further explored.

### LOX Expression Is Regulated by the PI3K/AKT, MEK/ERK, and SAPK/JNK Signaling Pathways

**FIGURE 4:**
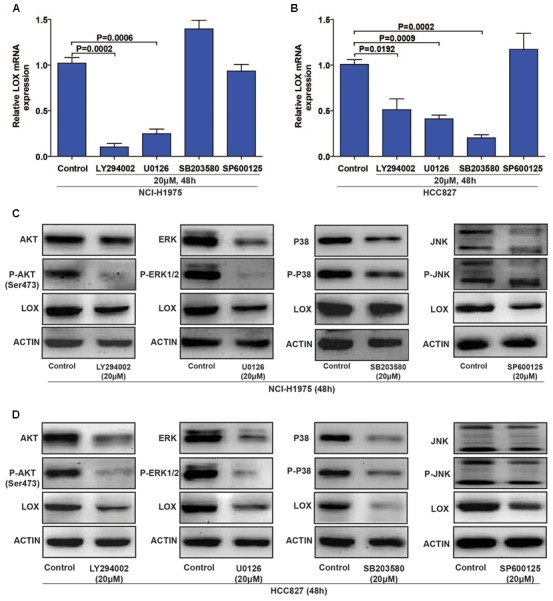
Lysyl oxidase expression is regulated by the PI3K/AKT, MEK/ERK, and SAPK/JNK signaling pathways. **(A,C)** NCI-H1975 was exposed to LY294002 (PI3K inhibitor), U0126 (MEK inhibitor), SB203580 (P-38 inhibitor) and SP600125 (JNK inhibitor) for 48 h, LOX mRNA and protein expression were analyzed by PCR and Western Blot. **(B,D)** HCC827 was exposed to LY294002 (PI3K inhibitor), U0126 (MEK inhibitor), SB203580 (P-38 inhibitor), and SP600125 (JNK inhibitor) for 48 h, LOX mRNA, and protein expression were analyzed by PCR and Western Blot. The data are represented as the mean ± SD. of three independent experiments. The *P*-values < 0.05 were considered statistically significant for all the tests.

To evaluate the regulatory mechanism by which LOX is regulated by EGFR, we focus on the four classical EGFR signal pathways (PI3K/AKT, MEK/ERK, P38 MAPK, and SAPK/JNK) with specific inhibitors (LY294002 for PI3K, U0126 for MEK, SB203580 for P-38, and SP600125 for JNK) (Keld and Ang, 2011). As shown in **Figures 4A,C** (**Supplementary Figures S3A–D**), LOX expression was dramatically down-regulated by PI3K/AKT and MEK/ERK inhibition at both the mRNA and protein levels in the NCI-H1975 cell, and the inhibition of the SAPK/JNK pathway also leads to the decreased expression of LOX protein. Moreover, when treated with the PI3K/AKT, MEK/ERK, and P38 MAPK inhibitors in another EGFR mutation NSCLC cell line HCC827, LOX expression at the mRNA and protein levels was inhibited. After the inhibition of the SAPK/JNK pathway, we obtained consistent data with the NCI-H1975 cells (**Figures 4B,D** and **Supplementary Figures S3E–H**). However, SAPK/JNK inhibition decreased the protein level of LOX but not mRNA level. This may because PI3K/AKT and MEK/ERK pathway regulate LOX expression through transcriptional level, while SAPK/JNK pathway via ubiquitin–proteasome system or lysosomal system. Next, we will investigate the underlying mechanism in the following study. Briefly, as is shown above, LOX expression was mainly regulated by the EGFR signal pathways PI3K/AKT, MEK/ERK, and SAPK/JNK in NSCLC cell lines.

### Silibinin Inhibits the Migration of the NSCLC Cell Line NCI-H1975 *in Vitro*

**FIGURE 5:**
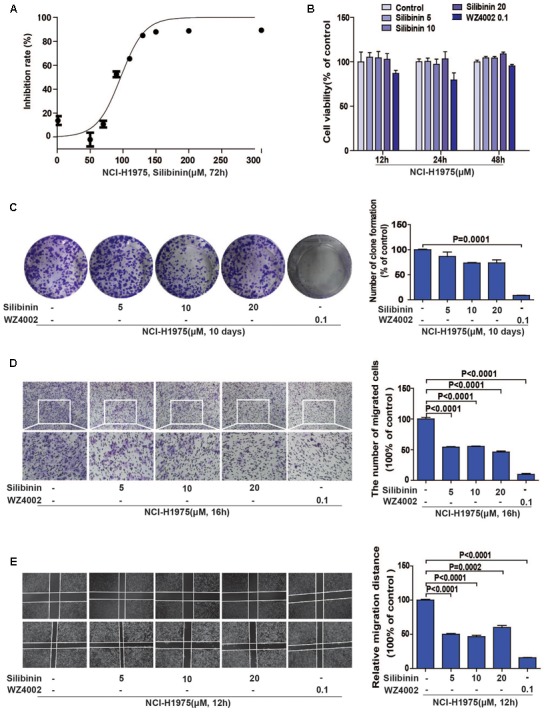
Silibinin inhibits the migration of the NSCLC cell line NCI-H1975. **(A)** MTT assay was performed to measure the IC_50_ of silibinin in NCI-H1975 for 72 h. **(B)** Silibinin treatment at concentrations of 5, 10, 20 μM for 12, 24, 48 h shows no anti-proliferative effect on the NCI-H1975 cells. WZ4002 treatment at 0.1 μM for 12, 24, and 48 h shows no anti-proliferative effect on the NCI-H1975 cells. **(C)** Clone formation assay was used to investigate the long-term anti-proliferative effect of silibinin or WZ4002 at the treatment concentrations. **(D)** NCI-H1975 cells migration in the transwell chambers was decreased after the treatment with silibinin or WZ4002 for 16 h. **(E)** NCI-H1975 cells migration was decreased after treatment with silibinin or WZ4002 for 12 h. The data are represented as the mean ± SD of three independent experiments. The *P*-values <0.05 were considered statistically significant for all the tests.

Accumulating evidences show that silibinin, a classical anti-fibrosis drug, exhibits strong anti-cancer activity in several human malignant tumors (Wu et al., 2013; Bosch-Barrera et al., 2016), but the underlying mechanism is still unknown. In consideration of one important molecule during fibrosis is LOX in hepatocyte, we suspected that whether the EGFR/LOX pathway plays a role in its anti-metastasis effect. Above all, we carried out an MTT assay to determine the effect of silibinin on the proliferation of NCI-H1975 cells, the IC_50_ was 96.56 ± 2.755 μM (**Figure 5A**). Meanwhile, the effect of silibinin on the migration of NCI-H1975 cells was measured by wound healing and transwell assay. As shown in **Figures 5D,E**, silibinin significantly inhibited the migration of NCI-H1975 cells but scarcely showed any proliferation inhibition effect at the treatment concentration (**Figures 5B,C**). Here, the data indicated silibinin significantly inhibited the migration of NCI-H1975 cells.

### Silibinin Inhibits the Migration of the NSCLC Cell Line NCI-H1975 through EGFR/PI3K/LOX Pathway *in Vitro*

**FIGURE 6:**
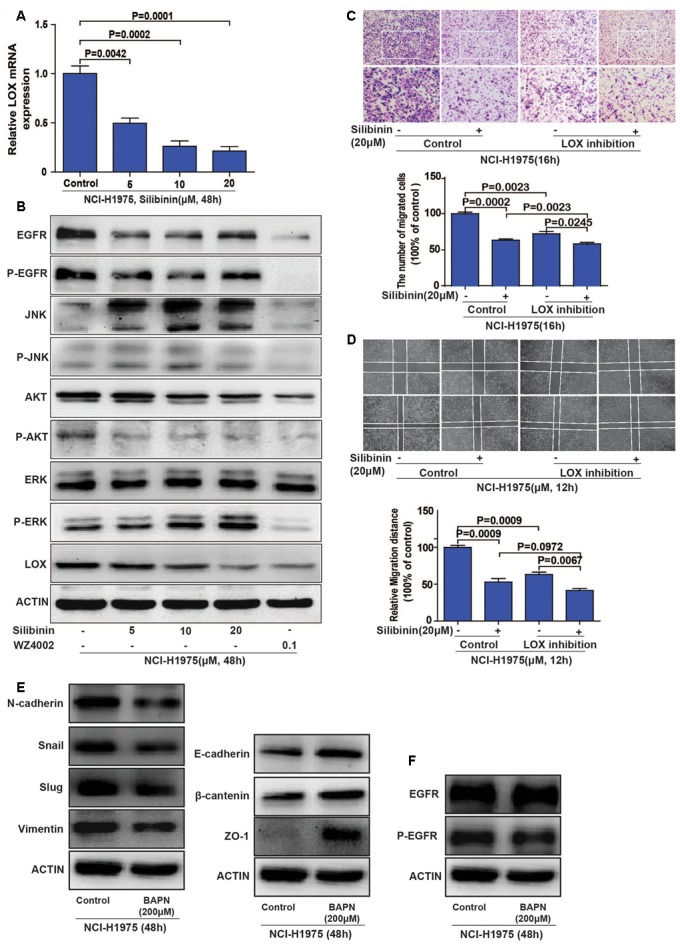
Silibinin inhibits the migration of the NSCLC cell line NCI-H1975 through the EGFR/PI3K/LOX pathway *in vitro*. **(A)** LOX mRNA expression after exposed to silibinin in NCI-H1975. **(B)** EGFR, P-EGFR, P-AKT and LOX expression were inhibited after the NCI-H1975 cells were treated with silibinin. **(C,D)** Pretreating the NCI-H1975 cells with the LOX inhibitor (BAPN 200 μM) decreased the anti-metastasis effect of silibinin. **(E)** LOX inhibition inhibited epithelial-to-mesenchymal transition of NCI-H1975. **(F)** LOX inhibition decreased the phosphorylation of EGFR but not the total expression of EGFR. The data is represented as the mean ± SD of three independent experiments. The *P*-values <0.05 were considered statistically significant for all the tests.

To explore if the EGFR/LOX signal pathway accounts for the anti-metastasis effect of silibinin, we investigated the relative signal pathways by PCR and Western Blot. The results showed that LOX mRNA expression was significantly decreased after silibinin treatment for 48 h (**Figure 6A**), silibinin also inhibited the EGFR/PI3K/LOX signaling pathway (**Figure 6B** and **Supplementary Figure S4**). Our above study shows PI3K/AKT, MEK/ERK, and SAPK/JNK pathways all participate in the regulation of EGFR to LOX. However, in consideration silibinin is a natural medicine, other signal molecules affected by silibinin may also act on p-JNK and p-ERK pathways. Meanwhile, our treatment dose is far below the doses in other researches (Bhatia and Falzon, 2015; Shukla et al., 2015). Thus, silibinin regulates LOX expression mainly through PI3K/AKT pathway in our treatment doses. When the LOX inhibitor (BAPN) blocked the migration of NCI-H1975 cells, it also showed that pretreatment with the LOX inhibitor attenuated the anti-metastasis effect of silibinin (**Figures 6C,D**). Though, silibinin is still able to exhibit its anti-metastasis effect maybe via other signal pathways after the inhibition of LOX. But the fold change of silibinin on migration after LOX inhibition is much smaller than it without LOX inhibition.

Meanwhile, cancer cells often change their cellular and molecular morphology during the metastasis process. Then we investigated the expression of epithelial-to-mesenchymal transition (EMT) molecules in NCI-H1975 cells treated with LOX inhibitor. The results showed that LOX inhibition supressed the expression of vimentin, slug, snail and N-cadherin, and increased the expression of epithelial phenotypic markers such as E-cadherin, ZO-1, and β-cantenin (**Figure 6E** and **Supplementary Figures S5A–G**), suggesting LOX inhibition could inhibit EMT in NSCLC cells. Coincidently, the previous study reported that silibinin could repress the EMT to suppress the invasive property of metastatic prostate cancer cells (Wu et al., 2010; Cufi et al., 2013). According to our result that silibinin could decrease the expression of LOX (**Figures 6A,B**), it suggested the inhibition of EMT by silibinin may be via LOX. In addition, the phosphorylation of EGFR was also decreased with the inhibition of LOX, but not the total expression of EGFR (**Figure 6F** and **Supplementary Figures S5H–I**), which indicating there may be a regulation loop between P-EGFR and LOX. In conclusion, silibinin exhibited an anti-metastasis effect possibly via LOX regulated EMT molecules. But whether silibinin inhibits migration by targeting EGFR is unknown.

**FIGURE 7:**
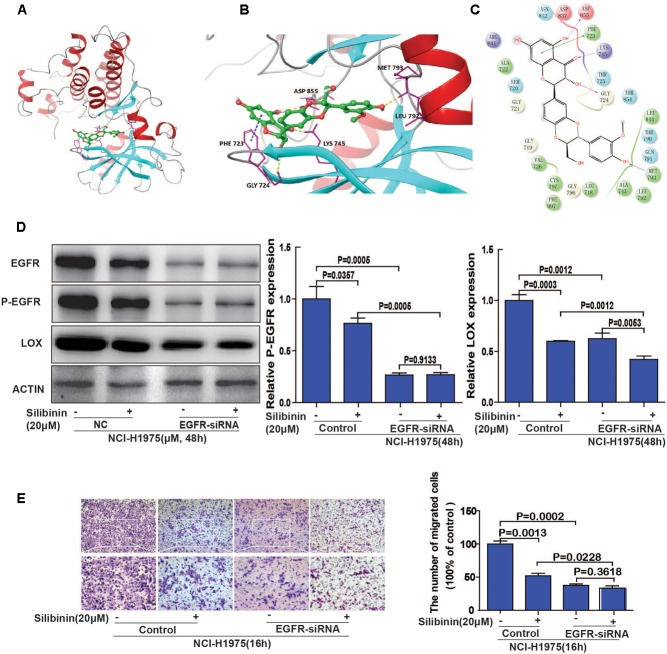
EGFR plays an important role in silibinin-attenuated NSCLC cell line NCI-H1975 migration. **(A)** The overall structure of silibinin binding to EGFR. **(B)** The detailed interactions (formation of four hydrogen bonds and 1 π–π stacking). **(C)** Two-dimensional map of silibinin binding to EGFR. **(D)** Effect of silibinin on LOX expression in EGFR knockdown NCI-H1975 cells. The NCI-H1975 cells were pretreated with EGFR siRNA for 8 h before the addition of silibinin (20 μM) for 48 h for the Western Blot analysis. **(E)** The effect of silibinin on EGFR knockdown NCI-H1975 cells migration. The NCI-H1975 cells were pretreated with EGFR siRNA for 8 h before the addition of silibinin (20 μM) for 16 h for the transwell assay. The data is represented as the mean ± SD. of three independent experiments. The *P*-values <0.05 were considered statistically significant for all the tests.

Molecular docking studies predict potential interactions of the proposed protein with the selected molecule, which is a structural modeling approach to study possible binding sites for cancer therapeutics. To explore the involvement of EGFR as a therapeutic target of silibinin, we first used molecular docking methods to predict the binding affinity of EGFR and silibinin. Based on the receptor structure combined with the docking pharmacophore virtual screening method with G-score (-11.069), silibinin formed four hydrophobic interactions (GLY 724, ASP 855, LYS 745, and MET 793) and 1 π–π stacking (PHE 723) with EGFR. This result suggests that silibinin could strongly bind to the functional domains of EGFR and affect the bioactivity of EGFR (**Figures 7A–C**). Then, as shown in **Figure 7C**, LOX expression was significantly decreased after treatment with silibinin and EGFR-siRNA. Meanwhile, the fold change of silibinin on migration after knockdown of EGFR is much smaller than it without knockdown of EGFR. These result suggested that knockdown of EGFR partially attenuated the effect of silibinin on LOX expression and migration (**Figures 7D,E**). In other words, silibinin exhibited anti-metastasis partly via targeting EGFR. Therefore, the data suggested that the EGFR/PI3K/LOX pathway might contribute to the anti-migration effect of silibinin.

### Silibinin Decreases NSCLC Metastasis via the EGFR/LOX Pathway *in Vivo*

**FIGURE 8:**
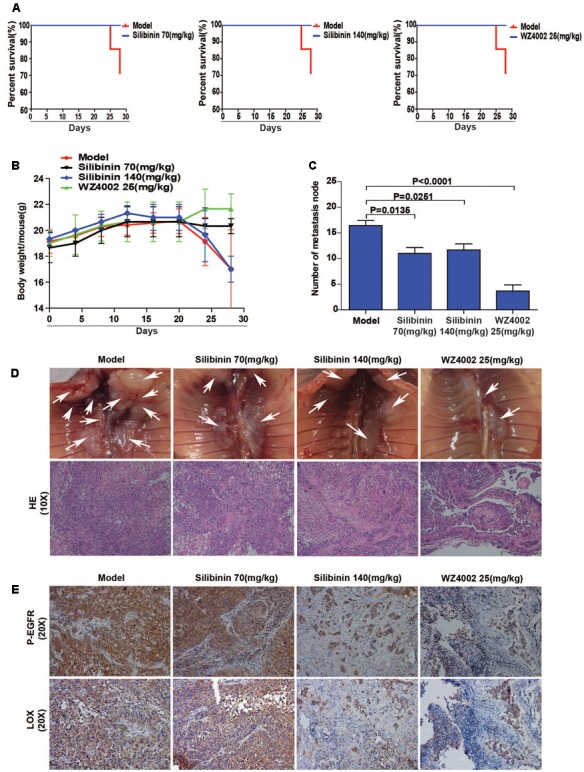
Silibinin decreases NSCLC metastasis via the EGFR/LOX pathway *in vivo*. **(A)** Silibinin treatment with 70 mg/kg and 140 mg/kg, WZ4002 treatment with 25 mg/kg prolonged the survival of the tumor-bearing mice. **(B)** Silibinin treatment with 70 mg/kg and WZ4002 treatment with 25 mg/kg improved the quality of life of tumor-bearing mice. **(C,D)** The metastasis nodes were decreased in the administration groups (silibinin 70 mg/kg, silibinin 140 mg/kg, WZ4002 25 mg/kg). **(E)** P-EGFR and LOX expression was decreased in in tumor tissues of the administration groups.

The regulatory relationship between the phosphorylation of EGFR and LOX, the inhibitory effect of silibinin on NSCLC cell migration was confirmed *in vitro*. However, whether silibinin inhibited NSCLC metastasis through EGFR/LOX *in vivo* remains unknown. In the orthotopic implantation metastasis model, we discovered that EGFR inhibitor WZ4002 and silibinin not only significantly prolonged the survival of the mice but also decreased the formation of metastasis nodes (**Figures 8A,C,D**). Moreover, after treatment with WZ4002 or silibinin, the body weight of mice was also increased (**Figure 8B**). Furthermore, the expression of LOX was inhibited, which is consistent with the inhibition of P-EGFR by the treatment with WZ4002 and silibinin *in vivo* (**Figure 8E**). In summary, as shown above, it was confirmed that silibinin decreased NSCLC metastasis via the EGFR/LOX pathway *in vivo*.

## Discussion

**FIGURE 9:**
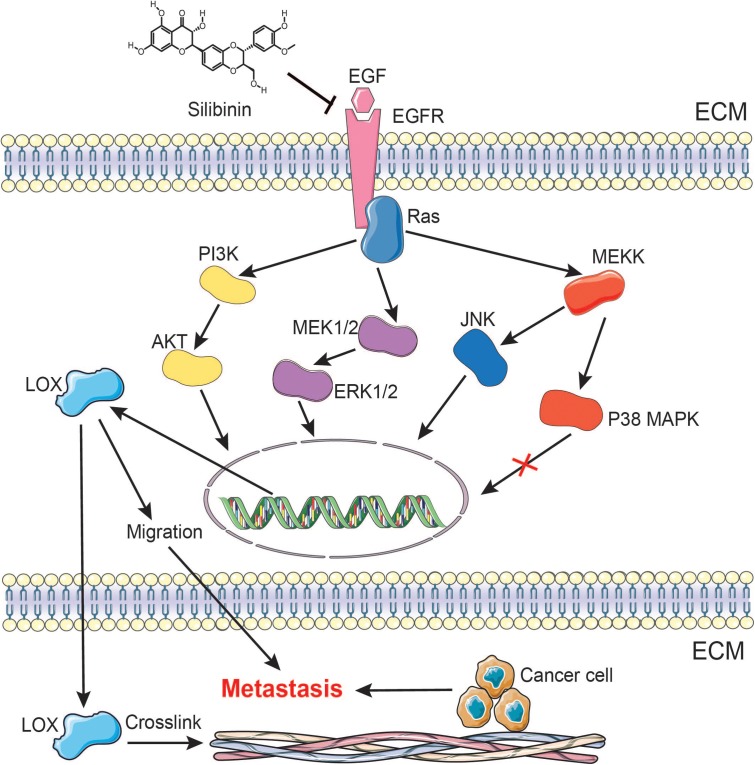
A schematic representation demonstrating the mechanism by which silibinin inhibits NSCLC metastasis via the EGFR/LOX signaling pathway.

Accumulating evidence suggests that LOX induces cancer progression by mediating a variety of steps in tumor metastasis (Rachman-Tzemah et al., 2017). However, the regulatory mechanism of LOX remains largely unexplored. Furthermore, silibinin, as an anti-fibrosis drug, exhibits the anti-metastasis effects in several cancers, but the mechanism is poorly understood. In this study, we confirmed the association between LOX expression and lung adenocarcinoma poor prognosis. Meanwhile, our data also showed that LOX was regulated by the EGFR pathway in NSCLC. In addition, we confirmed that the EGFR/LOX pathway might contribute to the anti-metastasis effects of silibinin (**Figure 9**). In this regard, our results elucidated a new pathway of the EGFR signaling and suggested the potential therapeutic target of LOX for the treatment in tumor metastasis. Importantly, our data revealed a novel mechanism that might provide a specific basis for the development of silibinin.

LOX may be a metastasis promoter in cancer progression. The involvement of the LOX is accepted as a potential poor prognostic factor for patients with cancer. It has only been reported that LOX is regulated by HIF-1α under hypoxia and is induced by TGF-β in cancer cells, the mechanisms are still largely uncovered. Studies on LOX previously focused on breast cancer (Wuest et al., 2015; Han et al., 2016). However, we found that LOX expression is more related to the poor prognosis of lung adenocarcinoma, as is shown in **Figure 1**. According to the epidemiologic study, EGFR mutation and consistent activation is an important marker of NSCLC poor prognosis, it covers more than half of the NSCLC patients (Jiang T. et al., 2016). Additionally, cancer cells get acquired resistance after about 6 months of TKIs treatment which is far beyond the speed of the development of new inhibitors. Thus, there is a pressing need to find a substitutable target in the EGFR pathway to overcome this drug resistance. Hence, we wondered whether there is a connection between LOX and EGFR. Interestingly, we found that LOX expression was regulated by EGFR via the PI3K/AKT, MEK/ERK and SAPK/JNK signaling pathways, suggesting that LOX might be a potential candidate for NSCLC therapy (**Figures 2**–**4**). At last, we found that LOX inhibition could dramatically inhibit the phosphorylation of EGFR but not the total expression of EGFR, which deserves further exploration (**Figure 6**).

Unfortunately, the development of a novel EGFR inhibitor is far from addressing rapid acquired resistance. The National Cancer Institute’s report showed that over 60% of the new approved anti-cancer drugs were natural products or derived from a natural product (Newman and Cragg, 2012). A number of researchers from all over the world are focusing on natural products to find novel anti-cancer drugs. Silibinin is an active constituent isolated from the seeds of Silybum marianum (Chang et al., 2015). Researchers previously paid more attention to the prevention and/or treatment of liver disorders (Stanca et al., 2013; Rendina et al., 2014). Recently, amount of evidence suggests that silibinin possesses pleiotropic anticancer effects in different cancers. According to clinical trial^8^, several clinical studies of silibinin for cancer therapy are in progress or have been completed. As reported, silibinin induces cancer cell cycle arrest (Feng et al., 2015), apoptosis (Zhang et al., 2015), inhibits tumor energy metabolism (Raina et al., 2013), significantly inhibits tumor metastasis (Bosch-Barrera and Menendez, 2015; Jiang C. et al., 2016). Importantly, Menendez’s study showed that lung cancer patients treated with silibinin had significantly inhibited brain metastasis in clinical (Bosch-Barrera et al., 2016). Silibinin also showed a therapeutic effect on bladder cancers (Wu et al., 2013), breast cancers (Kim et al., 2016), prostate cancers (Deep et al., 2014), liver cancers (Polachi et al., 2016), etc. However, the mechanism is poorly understood. We confirmed that the EGFR/LOX signalling pathway is involved in the anti-metastasis effects of silibinin (**Figures 5**–**8**).

Silibinin shows anti-cancer effect on EGFR TKI resistant cell NCI-H1975, which indicating silibinin may overcome the resistant to first generation TKIs (gefitinib and erlotinib). Importantly, according to our results from molecular docking, the binding site of silibinin with EGFR is different from the binding site of the third generation EGFR TKIs (such as osimertinib), which means silibinin may be also effective on the EGFR mutation cells with resistant to latest EGFR TKIs (osimertinib). Meanwhile, as an EGFR inhibitor, it is also interesting to investigate whether the combination of silibinin and other EGFR inhibitor (gefitinib and erlotinib) could overcome the drug resistance in cancer therapy. Coincidently, amount of tumor patients are accompanied with hepatic fibrosis or liver damage under chemotherapy. As a classical antifibrotic drug, silibinin is expected to develop into an extensive anti-cancer therapeutic strategy based on drug repurposing. In addition, researchers also report that other traditional drugs, such as metformin and pirfenidone, exhibit significant anti-cancer effects (Cheng et al., 2016; Mediavilla-Varela et al., 2016), drug repurposing can be a research direction in anti-cancer drug development as well.

Both our experiment (*in vivo* and *in vitro*) and reported clinical studies all confirmed the anti-cancer effect of silibinin, suggesting the possibility for clinical application. Unluckily, the metastasis does not affect with increasing dose of silibinin. The anti-cancer effect of silibinin in our lung orthotopic implantation metastasis model may have reached the best efficacy for the evaluation method of “internal metastasis nodes”, thus the increasing dose of silibinin may not affect the metastasis. Meanwhile, our treatment doses even the high-dose group *in vivo* still relatively lower than the reported doses, the increase of treatment dose may get better efficacy in our mouse model (Raina et al., 2013). In addition, silibinin was administered intragastrically in our study, the bioavailability may influence silibinin plasma concentration of silibinin in the high-dose group.

Our study proposed a new mechanism for silibinin in cancer therapy, provided a scientific basis for the development of silibinin in further clinical research. For further evaluation, we are preparing to confirm the therapeutic effect of silibinin on NSCLC via patient-derived xenograft (PDX) models. Since multiple steps are involved in the formation of tumor fibrosis (Semenza, 2016), PLOD2 and P4HA also mediate essential sections of metastasis (Gilkes et al., 2013; Du et al., 2017b). Their involvement in the anti-metastasis effect of silibinin also deserves further study.

In summary, our research confirmed that LOX expression is regulated by EGFR via the PI3K/AKT, MEK/ERK and SAPK/JNK pathways, which revealing a new pathway of the EGFR signaling in NSCLC. In addition, the study also showed that the EGFR/LOX pathway may play a role in the anti-metastasis effects of silibinin in NSCLC. Importantly, LOX as an important effector in tumor metastasis, may be a potential target for cancer therapy in the future.

## Author Contributions

XH, HD, XA, MZ, SY, and LiS defined the research subject and its aims, conceived and designed the experiments. XH, HD, XQ, LeiS, QZ, YW, and YaL conducted the experiments. XH, JX, LL, and YoL analyzed the data and wrote the paper.

## Conflict of Interest Statement

The authors declare that the research was conducted in the absence of any commercial or financial relationships that could be construed as a potential conflict of interest.

## Footnotes

1 https://clinicaltrials.gov

2 http://kmplot.com/analysis/

3 https://www.oncomine.org

4 http://cancergenome.nih.gov

5 http://kmplot.com/analysis

6 https://www.oncomine.org

7 http://cancergenome.nih.gov/

8 http://www.clinicaltrials.gov

## Supplementary Material

FIGURE S1 **(A–C)** The band intensity of **Figure 2D**.

FIGURE S2 **(A)** The band intensity of **Figure 3A**. **(B)** The band intensity of **Figure 3B**. **(C)** The band intensity of **Figure 3G**. **(D)** The band intensity of **Figure 3H**. **(E)** The band intensity of **Figure 3I**.

FIGURE S3 **(A–D)** The band intensity of **Figure 4C**. **(E–H)** The band intensity of **Figure 4D**.

FIGURE S4 **(A–G)** The band intensity of **Figure 6A**.

FIGURE S5 **(A–G)** The band intensity of **Figure 6E**. (H,I) The band intensity of **Figure 6F**.
