# Supplementary figures and images for: Silibinin Inhibits NSCLC Metastasis by Targeting the EGFR/LOX Pathway
Source: Front Pharmacol. 2018 Feb 8;9:21. doi: 10.3389/fphar.2018.00021 (PMC5809401; doi:10.3389/fphar.2018.00021)

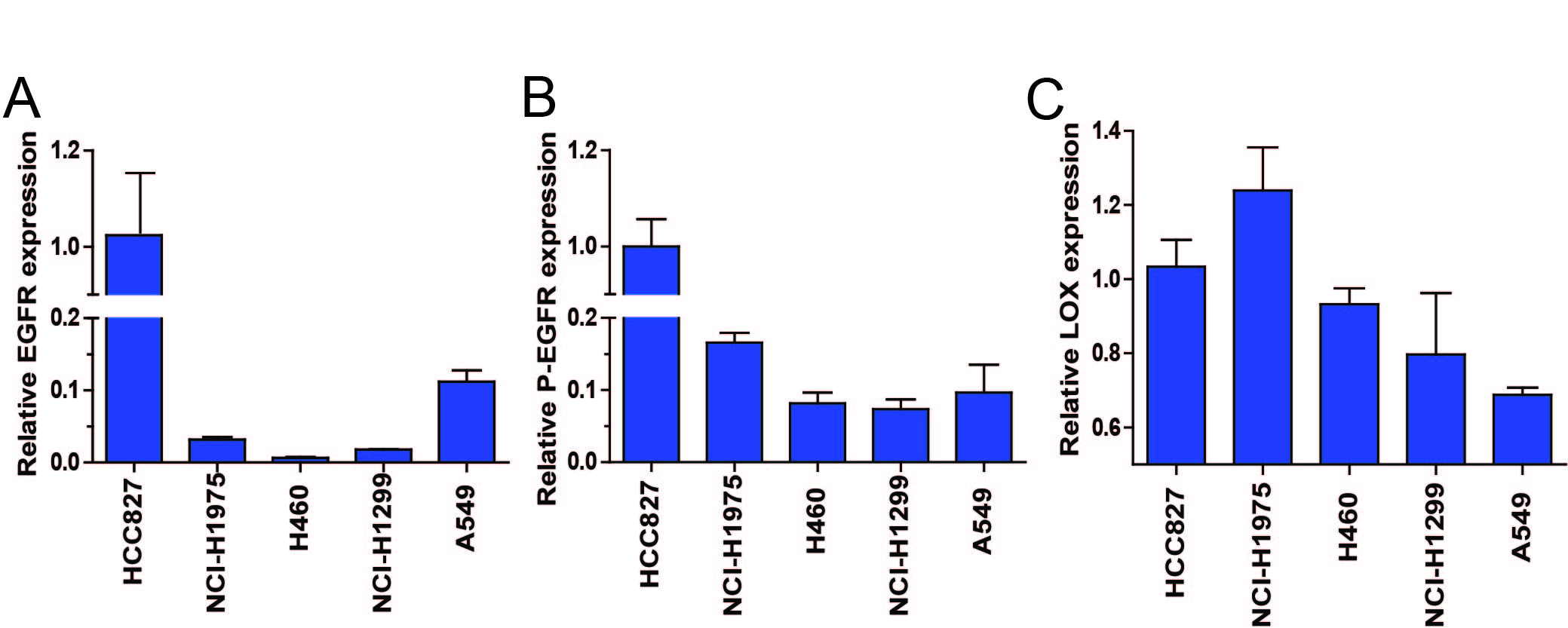

Supplement: FIGURE S1 — (A–C) The band intensity of Figure 2D. [file Image_1.JPEG]

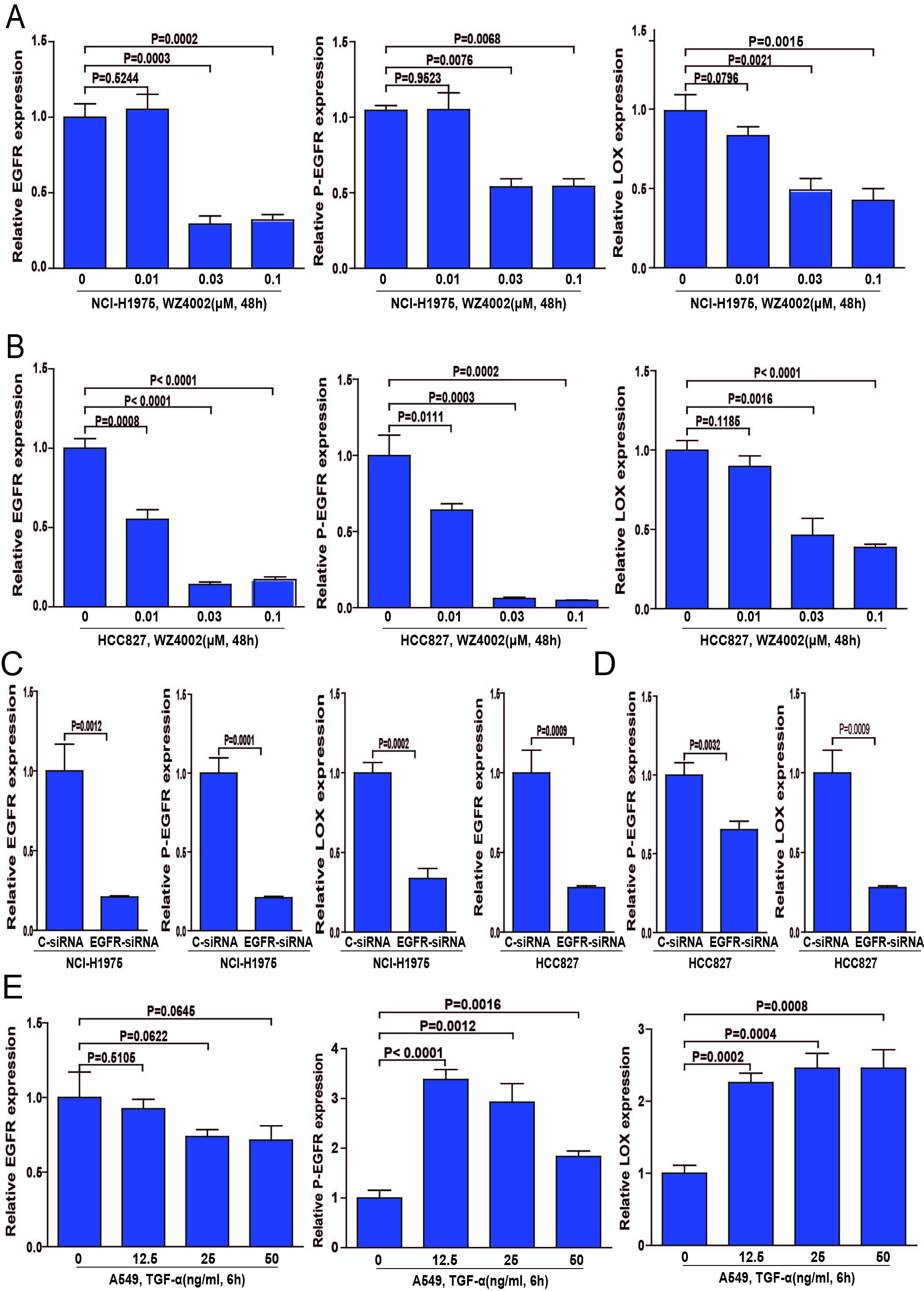

Supplement: FIGURE S2 — (A) The band intensity of Figure 3A. (B) The band intensity of Figure 3B. (C) The band intensity of Figure 3G. (D) The band intensity of Figure 3H. (E) The band intensity of Figure 3I. [file Image_2.JPEG]

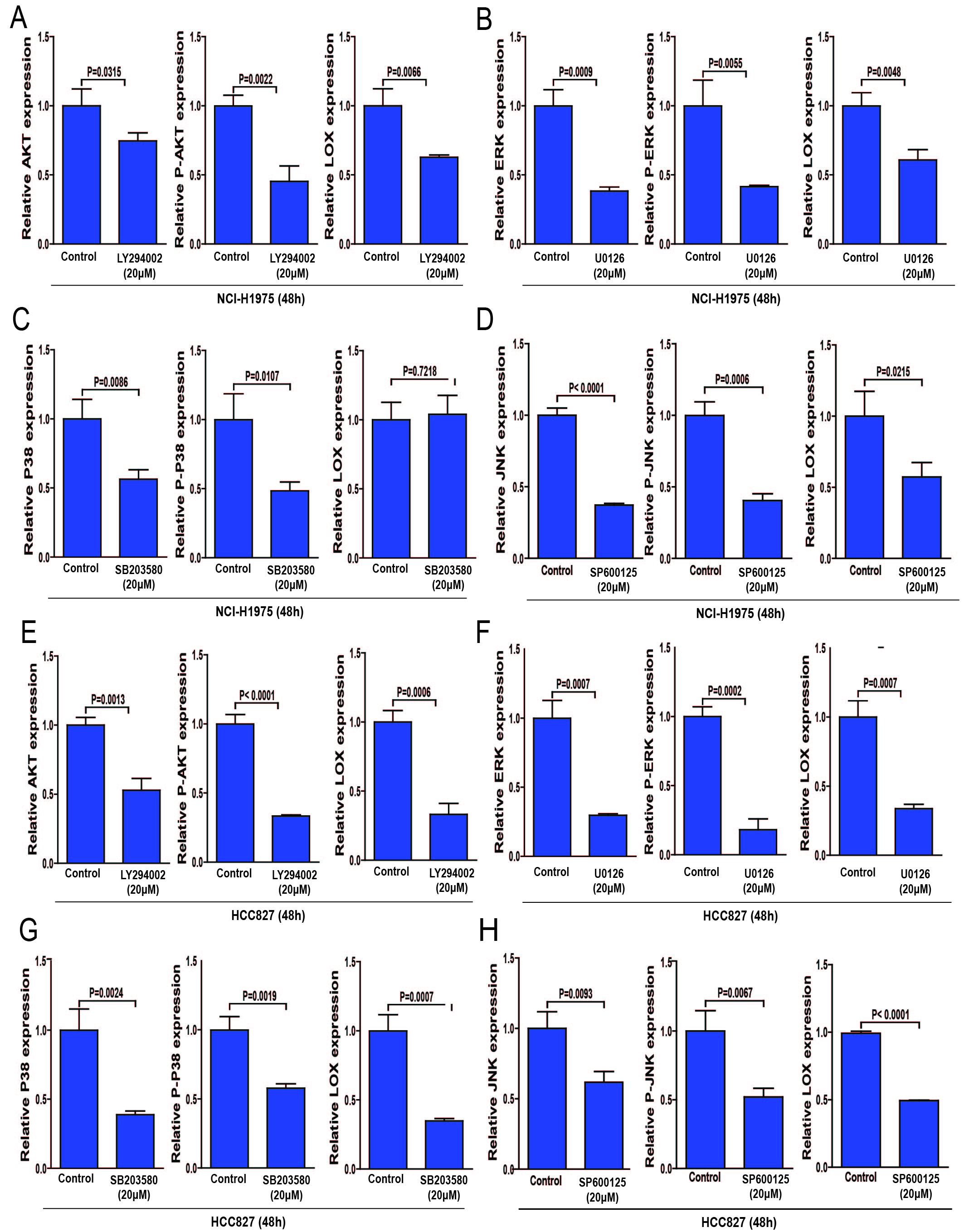

Supplement: FIGURE S3 — (A–D) The band intensity of Figure 4C. (E–H) The band intensity of Figure 4D. [file Image_3.JPEG]

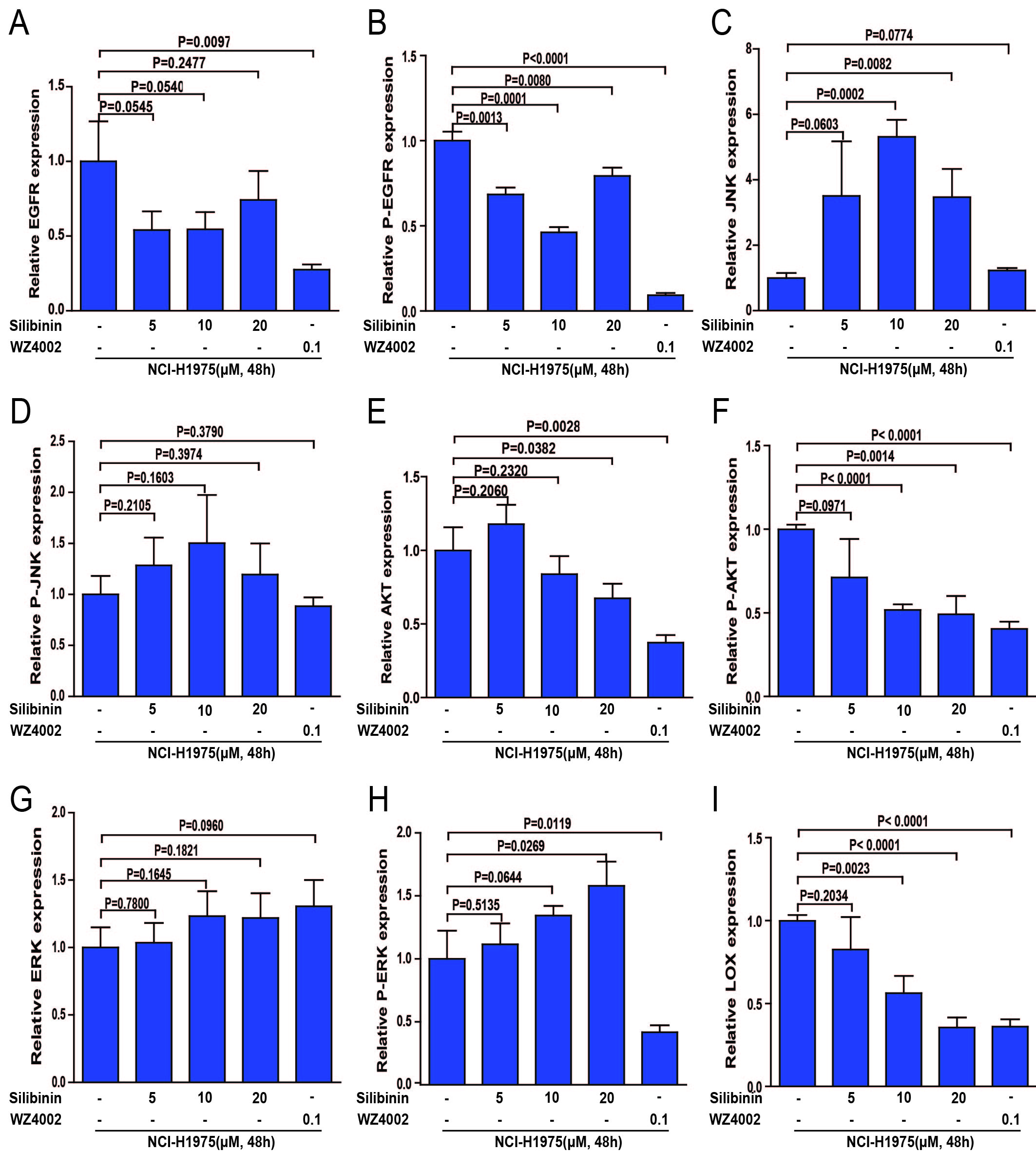

Supplement: FIGURE S4 — (A–G) The band intensity of Figure 6A. [file Image_4.JPEG]

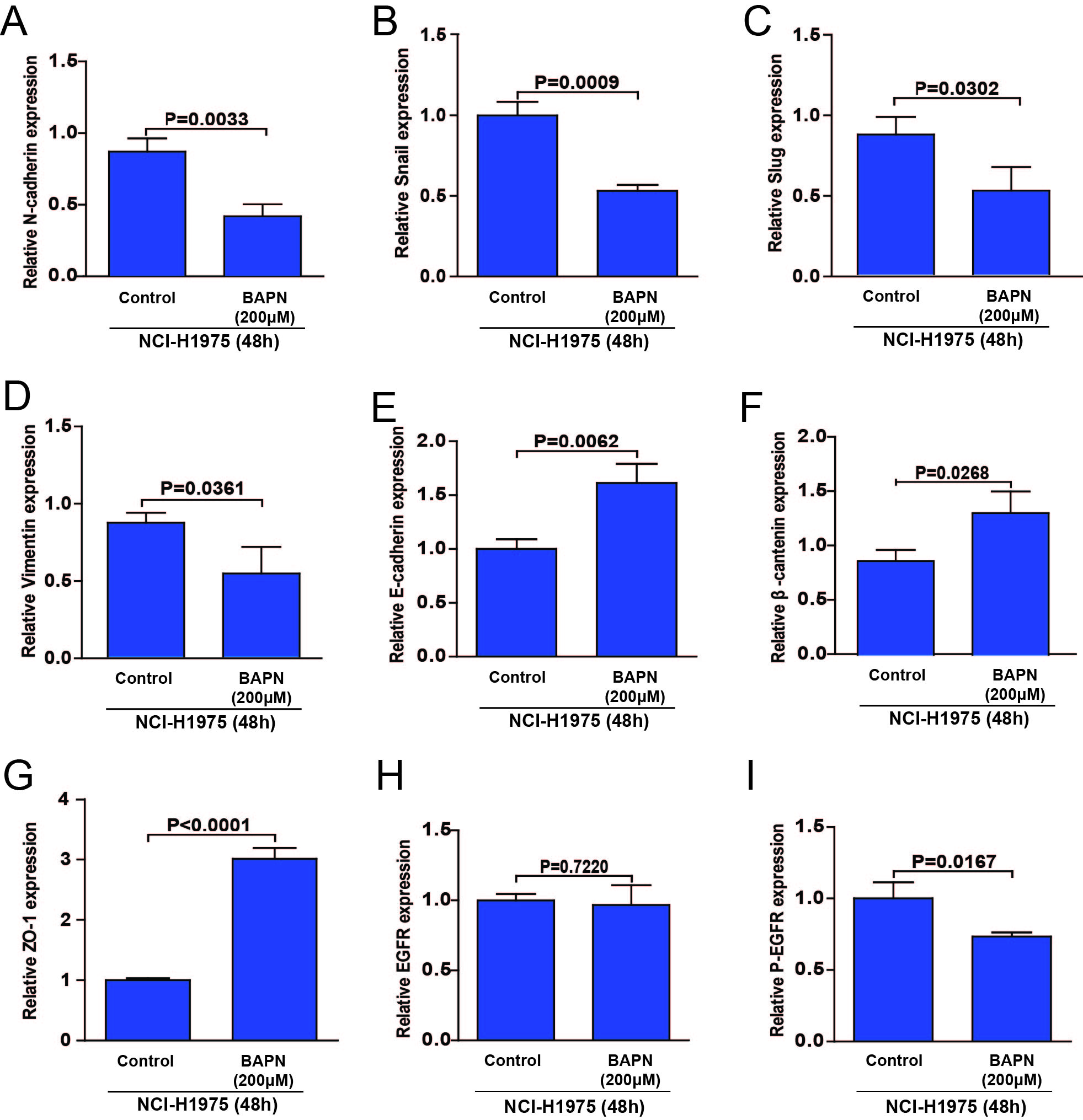

Supplement: FIGURE S5 — (A–G) The band intensity of Figure 6E. (H,I) The band intensity of Figure 6F. [file Image_5.JPEG]
